# A new perspective on how humans assess their surroundings; derivation of head orientation and its role in ‘framing’ the environment

**DOI:** 10.7717/peerj.908

**Published:** 2015-06-18

**Authors:** Gwendoline Ixia Wilson, Mark D. Holton, James Walker, Mark W. Jones, Ed Grundy, Ian M. Davies, David Clarke, Adrian Luckman, Nick Russill, Vianney Wilson, Rosie Plummer, Rory P. Wilson

**Affiliations:** 1 Swansea Lab for Animal Movement, Biosciences, College of Science, Swansea University, Singleton Park, Swansea, Wales, UK; 2 College of Engineering, Swansea University, Singleton Park, Swansea, Wales, UK; 3 Computer Science, College of Science, Swansea University, Singleton Park, Swansea, Wales, UK; 4 Department of Mathematics, College of Science, Swansea University, Singleton Park, Swansea, Wales, UK; 5 Department of Geography, College of Science, Swansea University, Singleton Park, Swansea, Wales, UK; 6 TerraDat, Cardiff, Wales, UK; 7 National Botanic Garden of Wales, Llanarthne, Camarthen, Wales, UK

**Keywords:** Environmental framing, Head attitude, Navigation behaviour

## Abstract

Understanding the way humans inform themselves about their environment is pivotal in helping explain our susceptibility to stimuli and how this modulates behaviour and movement patterns. We present a new device, the Human Interfaced Personal Observation Platform (HIPOP), which is a head-mounted (typically on a hat) unit that logs magnetometry and accelerometry data at high rates and, following appropriate calibration, can be used to determine the heading and pitch of the wearer’s head. We used this device on participants visiting a botanical garden and noted that although head pitch ranged between −80° and 60°, 25% confidence limits were restricted to an arc of about 25° with a tendency for the head to be pitched down (mean head pitch ranged between −43° and 0°). Mean rates of change of head pitch varied between −0.00187°/0.1 s and 0.00187°/0.1 s, markedly slower than rates of change of head heading which varied between −0.3141°/0.1 s and 0.01263°/0.1 s although frequency distributions of both parameters showed them to be symmetrical and monomodal. Overall, there was considerable variation in both head pitch and head heading, which highlighted the role that head orientation might play in exposing people to certain features of the environment. Thus, when used in tandem with accurate position-determining systems, the HIPOP can be used to determine how the head is orientated relative to gravity and geographic North and in relation to geographic position, presenting data on how the environment is being ‘framed’ by people in relation to environmental content.

## Introduction

Aristotle is reported to have said “sight…is the sense yielding the most knowledge and excelling in differentiation” (Jonas, 1954; Nasar, Hecht & Wener, 2008), so it is little surprising that such a substantial part of the human brain is used for processing visual information (Mishkin, Ungerleider & Macko, 1983). It is therefore of little surprise that vision plays such a pivotal role in structuring human perception of the environment (Hansen & Ji, 2010) and in affecting behaviour (Cerrolaza, Villanueva & Cabeza, 2012). Correspondingly, the physiology, anatomy and functioning of the eyes have been a subject of interest for decades (see e.g., Walls, 1942).

The pivotal nature of vision in studies examining how humans move and behave has led to research efforts that try to identify the visual attention given to objects within the environment (Arrington et al., 2000). What we actually see is determined by three things: body orientation, head attitude with respect to the body, and eye attitude within the head. Despite the relevance of all three, the most common method for investigating the natural performance of eyes is ‘eye tracking,’ which traditionally uses a camera to determine the orientation of the eye (Cleveland, Cleveland & Norloff, 1993) and produces metrics of natural eye movements such as ‘fixations’ and ‘scanpaths’ (Cooke, 2005). Improvements in eye-tracking type techniques have now resulted in a plethora of publications which have allowed characterisation of eye movement, and examination of perceptual span and eye movement control (Rayner, 1998) and show the power of this rapidly expanding field (Duchowski, 2002).

Eyes can, however, only track objects if the head is so orientated that the objects are within the potential field of view (primarily determined by the functional range of eye movements (Stahl, 1999, EBR). In short, normal head attitude and head movements (Munhall et al., 2004) are expected to play a major role in defining the sectors of the environment in which people find elements for visual attention (Morasso, Bizzi & Dichgans, 1973). Indeed, specifically, head attitude is critical in ‘framing the environment’ and its consideration should therefore be of interest to studies seeking to determine the factors that lead to the details of how we actually do this. In particular, we would expect features of the environment to modulate attitudes adopted by the head (head pitch and head heading) because the head is expected to turn to centre the gaze on objects of interest while, at the same time, people will presumably only be able to react visually to elements that are within the visual field provided by the head attitude.

We propose an avenue of looking at this complex topic by reporting on the design and first use of a new system to track human head movement, the Human-Interfaced Personal Observation Platform—HIPOP. This unit is based on the use of a GPS-enabled Daily Diary (Wilson, Shepard & Liebsch, 2008) which is a device containing a carefully selected suite of sensors including accelerometers and magnetometers. The unit is fixed to an easily worn headmount and can be used to gauge the head orientation of human participants in a natural setting. We trialed the use of the HIPOP on people walking along a featureless corridor and in a botanical garden and described first results, suggesting reasons for observed patterns of head attitude.

## Method

### Device design

**Figure 1:**
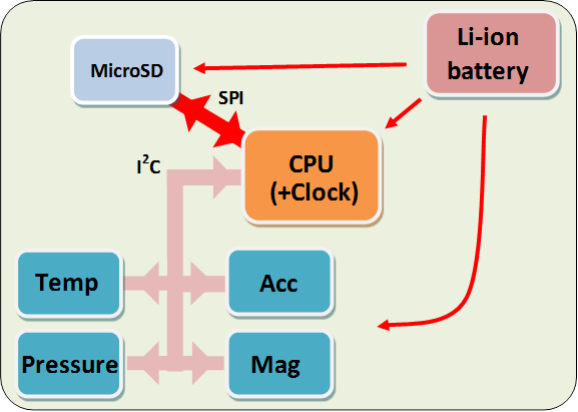
Schematic diagram of the construction of the HIPOP showing the constellation of the main components.

**Figure 2:**
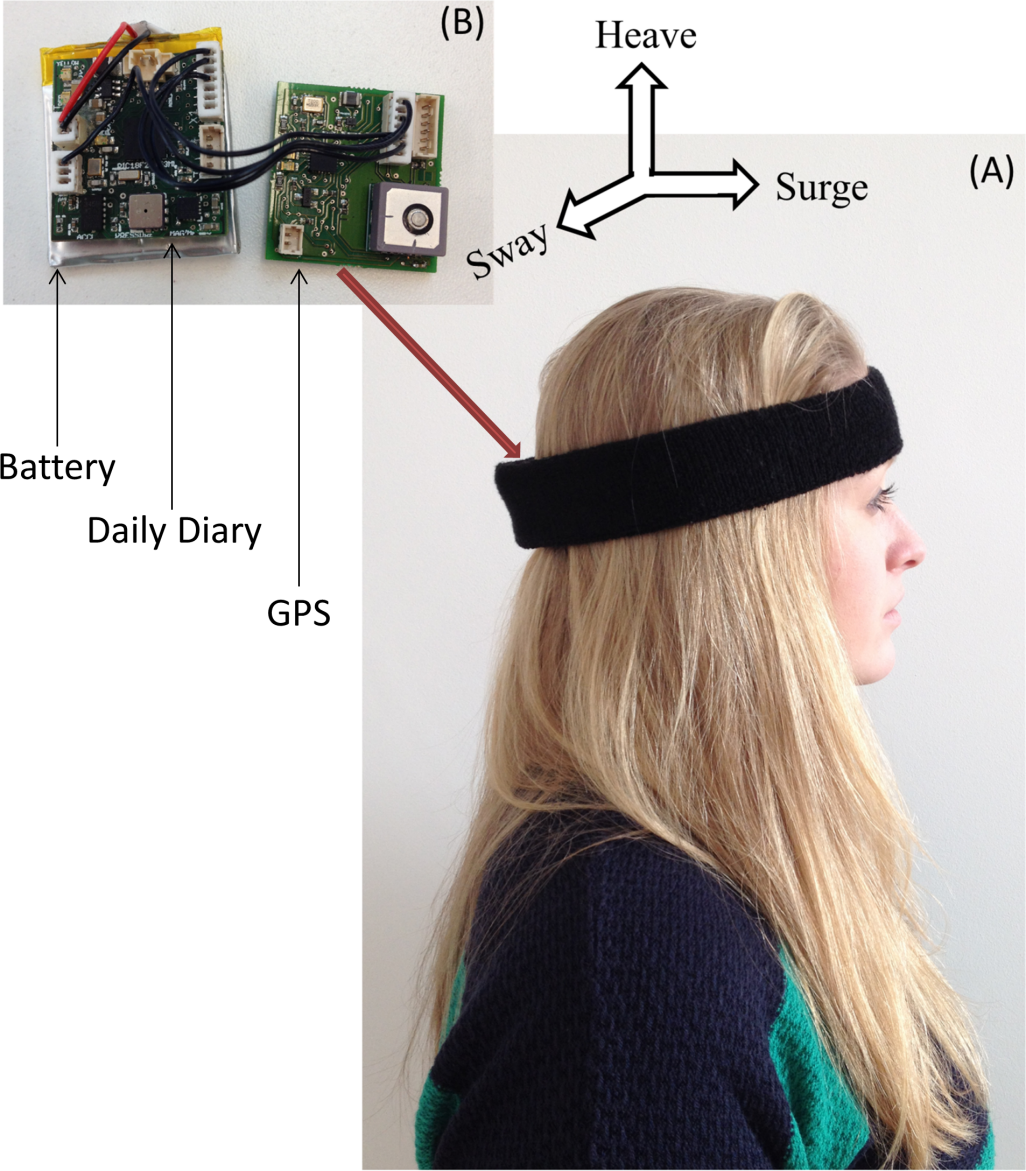
Image of participant wearing headmount containing HIPOP. (A) The red arrow shows the tags positioned in the headband and (B) shows the HIPOP consisting of the Daily Diary, GPS and the battery outside the casing.

The HIPOP consists of a GPS-enabled Daily Diary (DD) (Wilson, Shepard & Liebsch, 2008) (supplier Wildbyte Technologies— http://www.wildbyte-technologies.com/) composed of two circuit boards, one of which carries the GPS module (26 × 27 × 5.5 mm–mass 2.5 g) using the Origin 1410 GPS and another, which consists of the main circuit board for the Daily Diary complete with sensors, processor (Microchip PIC18F26J53 with built-in real-time clock) and memory (Sandisk MicroSD −1 or 2 Gb) (26 × 27 × 9 mm–mass 3.2 g). The sensors on the Daily Diary are; a tri-axial accelerometer (Analog ADXL345), a tri-axial magnetometer (Honeywell HMC5883L), and a combined barometric pressure and temperature module (Bosch BMP085) (Fig. 1). Both boards are powered by a single rechargeable 300 mAh battery and packed into an L-shaped package, 3-d printed in PLA (polylactic acid) plastic so that the orientation of the main circuit board can be held perfectly/firmly against the back of the head (Fig. 2). During operation, the HIPOP draws between 7 and 35 mA, depending on the sampling frequency of the GPS, and so can be deployed with this battery for periods between 8 and 42 h.

### Mode of functioning

The basic premise behind the operation of the system is that, once calibrated in position on a participant’s head, it stores data that can be used to determine the head pitch angle (using the accelerometers) and the head orientation angle with respect to magnetic North (using data from the accelerometers and the magnetometers). These two parameters together can be used to determine precise head orientation. Finally, this head orientation is put into an environmental context by having the system coupled to a GPS, which gives the geographic position of the wearer.

During normal operation, the GPS module obtains an initial fix and determines the current time according to GMT/UTC. The Daily Diary then logs data at the user-specified frequency, typically 40 Hz, for the on-board sensors (accelerometer, magnetometer, temperature, and pressure), whilst the GPS circuit logs coordinates and time at 1 Hz, if reception allows. At the end of the logging session, the DD downloads the GPS coordinates and time-stamped data and stores them in its own data-store. Although the two devices do not log at the same frequency, the data are synchronised by the use of real-time clocks initialised at power-up, and kept synchronised by a crystal oscillator to eliminate clock-drift.

### Calibration and calculation of pitch and heading

#### Calibration of the device

All devices used within the study were first calibrated for response to magnetic field strength by mounting them on a flat surface with respect each of their 3 axes and rotating them through 360°. The magnetic dip angle at the calibration site was then used in regressions of angle *versus* sensor reading of each of the three magnetometer axes to describe the (linear) relationship before normalizing it for use in calculation of device heading (see below). The manufacturer of the magnetometer HMC5883L (Honeywell, Morristown, New Jersey, USA) specify a heading accuracy of between 1 and 2 degrees for the 12-bit output from the tri-axial, orthogonal, sensors. The device itself is set for a range of ± 0.88 Ga. Due to the variable nature of the magnetic field across the earth’s surface, it is important to establish the nature of the field in both strength and direction/polarity for all 3 axes at the point of data collection. This is done by simultaneously recording accelerometry data to determine device orientation (see above).

Tri-axial, orthogonal data from the accelerometer, were recorded with a range of ± 16 g, and a resolution of 4 mg, although in our use this was modulated by how the system was mounted on the head and the calibration on the user (see below).

#### Determination of head pitch angle

The 3 accelerometers within the HIPOP measured in orthogonal axes that were placed within the tag so that, once mounted on a participant’s head, they measured acceleration in the surge, heave and sway axes (Fig. 2). Following deployment, the raw acceleration data from the surge axis were smoothed over a 2 s window (80 points) to derive the static acceleration (that derived from gravity and the device posture) and omit the acceleration due to the linear displacement of the head (Shepard et al., 2008a; Shepard et al., 2008b). Thus, the surge axis nominally read 0 g when the HIPOP wearer stood upright and looked horizontally, the arcsin of which gives the device angle in degrees (Wilson, Shepard & Liebsch, 2008).

#### Determination of device heading

Determination of the HIPOP heading followed methods detailed in previous work (Fang et al., 2005; Freescale Semiconductor, 2012) and the Memsense technical report. The 3 magnetometers within the HIPOP aligned in orthogonal axes measured the strength and direction of the earth’s magnetic field. The earth’s magnetic field is approximate dipole, consisting of field lines originating at a point near the magnetic South Pole and terminating at a point near the magnetic North Pole. The strength and direction of field lines recorded by a tri-axial magnetometer sensor was used to determine compass headings.

The earth’s rotational axes where they meet the earth’s surface define the geographic north and south poles which are commonly used for navigational purposes. This is not to be confused with true magnetic north and south, which defines the points on the earth surface where the magnetic field lines initiate and terminate. The raw magnetometer readings could be used to derive heading relative to magnetic north and, to compensate for this, the declination angle was added to the derived heading to provide an estimate of the heading relative to geographic north. The declination angle for a geographic location can be accessed at http://www.ngdc.noaa.gov/geomag-web/#declination.

The magnetometers readings of the earth’s magnetic field can be subject to distortion and, typically, there are two primary sources of error in computing the heading; hard iron and soft iron. Hard iron deposits produce a magnetic field which causes a constant bias in the output. Examples of this occur in electrical equipment, such as a loudspeaker, or magnetized iron. Conversely, soft iron deposits are caused by ferrous materials which are more permissive to the magnetic field. The field lines extend through the ferrous materials and distort or stretch the magnetic field. Hard iron distortions tend to have a much larger contribution towards errors (Cai, Andersen & Malureanu, 2001). To compensate for these errors, we undertook a calibration procedure prior to the deployment. The device was rotated through 360° whilst horizontal to the earth and then rolled 90° and rotated again through 360°. This procedure allowed us to determine the mapping of magnetometer readings to heading for each axis with respect to North, East, South, and West.

Due to the nature of the HIPOP and the attachment position of the device, we could not assume that the device was horizontal throughout the study so we used a tilt-compensation to adjust for offsets from the horizontal plane. This was achieved by projecting each measurement from the magnetometer to a horizontal coordinate frame where the compass readings could be computed. The accelerometer attribute was used to determine the pitch and roll offsets to be applied.

Following deployment, the heading was derived from the raw magnetometer data. This was undertaken in several processing steps to identify the tilt-compensated heading with respect to geographic north while considering sources of error from hard and soft iron bodies. The steps for this were as follows;

#### Alignment correction

The three axes of the accelerometer were aligned with the three axes of the magnetometer axes, such that the corresponding *x*, *y* and *z* axes were all in the same direction.

#### Pitch and roll computation

Tilt-compensating the magnetometer output required the device orientation to be known with respect to pitch and roll. The device orientation was computed directly from the static component of the raw acceleration component by passing the raw acceleration with a moving mean over 2 s (Shepard et al., 2008b). We can compute the static acceleration for a sample *S_i_* given a window size *w* as the formula below:

\begin{eqnarray*} {S}_{i}=\frac{1}{w}\sum _{j=i-\frac{w}{2}}^{i+\frac{w}{2}}{S}_{j}. \end{eqnarray*}

The pitch (*θ*) and roll (0̸) were derived by taking the arctangent of the corresponding static *S_x_*, *S_y_* and *S_z_* channels of the accelerometer recordings.

\begin{eqnarray*} \mathrm{Roll}(\gamma )=(\mathrm{atan}2({S}_{x},\sqrt{{S}_{y}\bullet {S}_{y}+{S}_{z}\bullet {S}_{z}})\frac{180}{\pi } \end{eqnarray*}

\begin{eqnarray*} \mathrm{Pitch}(\beta )=(\mathrm{atan}2({S}_{y},\sqrt{{S}_{x}\bullet {S}_{x}+{S}_{z}\bullet {S}_{z})}\frac{180}{\pi }. \end{eqnarray*}

#### Hard iron correction

Since hard iron deposits cause a constant bias in the magnetometer recordings, a mapped flat rotation (*x* and *y* axis) to a two-dimensional plane, and the side rotation (*y* and *z* axis) to a separate plane, no hard iron deposits should produce a circle centred around the origin (0, 0) in both instances. Where hard iron distortions were found, to shift the centre of the circle away from the origin, we corrected by finding the minimum and maximum values for each magnetometer channel (*m_x_*, *m_y_* and, *m_z_*) during the calibration periods to compute the offset of the magnetometer values so they produce a circle centred about the origin. The following formulae compute the hard iron corrected magnetometer values (${m}_{x}^{{\prime}}$, ${m}_{y}^{{\prime}}$ and ${m}_{z}^{{\prime}}$):

\begin{eqnarray*} {O}_{x}=\frac{\max ({B}_{x})+\mathrm{min}({B}_{x})}{2} \end{eqnarray*}

\begin{eqnarray*} {O}_{y}=\frac{\max ({B}_{y})+\mathrm{min}({B}_{y})}{2} \end{eqnarray*}

\begin{eqnarray*} {O}_{z}=\frac{\max ({B}_{z})+\mathrm{min}({B}_{z})}{2} \end{eqnarray*}

\begin{eqnarray*} {m}_{x}^{{\prime}}={m}_{x}-{O}_{x} \end{eqnarray*}

\begin{eqnarray*} {m}_{y}^{{\prime}}={m}_{y}-{O}_{y} \end{eqnarray*}

\begin{eqnarray*} {m}_{z}^{{\prime}}={m}_{z}-{O}_{z}. \end{eqnarray*}

#### Normalization of the compass data

Although our magnetometers were calibrated to give values in Gauss, the values recorded for each axes in the magnetometer sensor may be different for the same measurement in all magnetometer systems and different devices may also vary in sensitivity. To compensate for this, the hard iron correct magnetometer channels (${m}_{x}^{{\prime}},{m}_{y}^{{\prime}}$ and ${m}_{z}^{{\prime}}$) can be normalized by the magnitude length, such that the magnitude information from the magnetometer vector is removed while maintaining the important directional component. The following formulas are used to obtain the normalized magnetometer channels (${m}_{x}^{{\prime\prime}},{m}_{y}^{{\prime\prime}}$ and ${m}_{z}^{{\prime\prime}}$):

\begin{eqnarray*} {f}_{m}=\sqrt{m{x}^{2}+{m y}^{2}+{m z}^{2}} \end{eqnarray*}

\begin{eqnarray*} {m}_{x}=\frac{{m}_{x}}{{f}_{m}} \end{eqnarray*}

\begin{eqnarray*} {m}_{y}=\frac{{m}_{y}}{{f}_{m}} \end{eqnarray*}

\begin{eqnarray*} {m}_{z}=\frac{{m}_{z}}{{f}_{m}}. \end{eqnarray*}

#### Coordinate frame adjustment

Ideally, the device should be level with the earth’s surface to determine headings. To account for the variability in the device orientation (potential head pitch and roll), the magnetometer coordinate frame was projected onto the horizontal. Here, we obtained the device orientation from the pitch and roll derived from the accelerometer attributes. Each normalized hard iron corrected magnetometer channel $r{m}_{x}^{{\prime\prime}},{m}_{y}^{{\prime\prime}}$, and ${m}_{z}^{{\prime\prime}}$ was rotated by the inverse of the pitch (*θ*) and roll (0̸) to give the rotated column vector *m^r^*.

\begin{eqnarray*} {R}_{x}(\theta )=\left(\begin{array}{ccc} \displaystyle 1&\displaystyle 0&\displaystyle 0\\ \displaystyle 0&\displaystyle \cos \theta &\displaystyle -\sin \theta \\ \displaystyle 0&\displaystyle -\sin \theta &\displaystyle \cos \theta \end{array}\right) \end{eqnarray*}

\begin{eqnarray*} {R}_{y}(\emptyset )=\left(\begin{array}{ccc} \displaystyle \cos \emptyset &\displaystyle 0&\displaystyle -\sin \emptyset \\ \displaystyle 0&\displaystyle 1&\displaystyle 1\\ \displaystyle \sin \emptyset &\displaystyle 0&\displaystyle \cos \emptyset \end{array}\right) \end{eqnarray*}

\begin{eqnarray*} {m}^{r}={R}_{x}(\theta ){R}_{y}(\emptyset )\left(\begin{array}{c} \displaystyle {m}_{x}\\ \displaystyle {m}_{y}\\ \displaystyle {m}_{z} \end{array}\right). \end{eqnarray*}

#### Soft iron correction

Soft iron deposits affect the magnetic field by distorting and stretching the magnetic field and compensation for this used the same calibration procedure, but based derivatives on the *x* and *y* tilt-compensated magnetometer values because soft iron only affects the direction rather than the strength of the field. Where there are soft iron deposits, plotting the calibration cycles into the two-dimension plane showed what should be a circle distorted into an ellipse. The basis of the soft iron correction was to determine the minor and major axes of the ellipse, corresponding to the long and short dimensions, respectively. These values defined the coordinate system for the ellipse, which were rotated by the angular offset of the minor and major axes to transform them to the global coordinate system and into a circle. This procedure is detailed in the Memsense technical report and outputs soft iron corrected magnetometer channels defined as: ${m}_{x}^{s},{m}_{y}^{s}$, and ${m}_{z}^{s}$.

#### Heading derivation

Compass heading (*H*) was determined using the *x* and *y* axes of the tilt-corrected hard and soft iron magnetometer components. The arctangent was applied to the ${m}_{x}^{s}$ and ${m}_{y}^{s}$ readings to determine the heading with respect to magnetic north. Adding or subtracting the declination angle resulted in the heading with respect to geographic north.

\begin{eqnarray*} H=(\mathrm{atan}2({m}_{y},-{m}_{x}))\bullet \frac{180}{\pi }. \end{eqnarray*}

Determination of head heading also suffered from inaccuracies due to imperfect placement of the HIPOP on the wearer’s head. This was corrected in a manner similar to that used for correcting head pitch, by asking participants on a defined spot (outside—to negate any spurious magnetic fields caused by buildings) to fixate on three objects at known headings and correcting the HIPOP-derived heading values accordingly.

### Tests on participants

We conducted two types of test: One was conducted on a convenience sample of 15 participants, 8 of which were female and 7 male (Mean age = 28) to examine the general operation and validity of the system within and around Swansea University. A second test was conducted on 27 participants between the ages of 18–60 in a green, outdoor setting, the National Botanic Garden of Wales (51°50′23.46″N, 4°9′4.74″W), located near Llanarthney, South Wales. Average age of this group was calculated to be 44.6, using 10 year bins of estimated age (SD = 13.5). Of these participants, 19 were male and 8 female. Approval for all procedures was granted by Swansea University Biosciences Ethics Committee (BH-001-2014). All participants gave written consent. No specific characteristics were required of participants though all had normal or corrected-to-normal vision. All participants submitted some information about themselves, including age and gender. They were subsequently equipped with a HIPOP fitted into an L-shaped casing to allow easy and unhindered use of these devices, affixed to the back of headwear (Fig. 2). Headwear offered was a cap or a headband.

#### Derivation of head pitch error during calibrations

Each participant equipped with a HIPOP was asked to fixate on a spot on a wall at 1 m distance from the eyes, at exactly eye height. The subject was then asked to fixate for a short period on two other points, 1 m directly above and 1 m directly below the first point, while maintaining the same position. Subsequently, the surge acceleration data (cf. Fig. 2) recorded by the HIPOP were smoothed over 2 s (see above) to give the value of the surge acceleration corresponding to head pitch in these three head pitch positions which should have corresponded to −45°, 0° and 45°(assuming that the eyes remain immobile within the eye sockets—see later). An estimation of the actual HIPOP pitch angle could then be converted into head angle by simple linear regression of the device pitch angles with the known head pitch angle and correcting the device angles to accord. The quality of the fit indicated the variance in this procedure. Multiple trials where participants were asked to fixate on the three spots at various intervals were conducted to allow us to assess this variability.

This procedure was repeated using the same participants but by asking them to wear glasses that constricted their vision by only allowing them to look through a 2 mm wide horizontal slit, placed exactly horizontally in front of their pupils. It was anticipated that this procedure would reduce variability in the pitch calibration procedure.

#### Derivation of derived head orientation during normal outside behaviour

Participants were fitted with a HIPOP, calibrated for pitch and heading (see above), and asked to move within a university quadrangle measuring approximately 36 × 46 m and containing no people. Here, they were asked to fixate on specific points of interest (A4 sheets of card with large numbers on them and placed around the quad at various distances (6.8 to 36 m), headings (5–352°) and elevations (−14° to 58° pitch)) so as to define overall ‘head orientation’ errors and variance. The subjects were allowed to navigate around the quadrangle before periodically being asked to stand on defined points on the ground marked with chalk whereupon they were instructed to fixate on 9 different cards around the quadrant for defined periods ranging between 1 and 10 s. The real angles of pitch and headings from the defined spots on the ground, from respective eye heights, to the card markers were determined using a Leica Vector 21(Vectronix, Switzerland) so that derived head angles and direct angles could be compared.

#### Derivation of broad patterns of head movement during normal travel

Following calibration (see above), participants were asked to walk in different settings so as to derive preliminary data on head movements in different environments. Environments were; (i) a white corridor, featureless except for unmarked doors leading to adjacent rooms (ii) outside in a green environment and (iii) outside but within a university setting (Swansea University).

## Results

### Overall system tests

#### Head pitch error

The calibration procedure using no mechanism to restrict vision by constraining eye movement within the eye orbits via glasses showed that head movement accounted well for vision fixation direction in terms of head pitch, with standard deviations from different subjects varying between a minimum of 2.6° and a maximum of 12.4° (overall mean SD = 6.4°). However, use of glasses to constrict eye movement reduced the variation appreciably to give minimum and maximum individual standard deviations ranging between 0.7° and 8.7° (overall mean SD = 2.8°), and thus a markedly better estimate of head directionality.

#### Overall error

**Figure 3:**
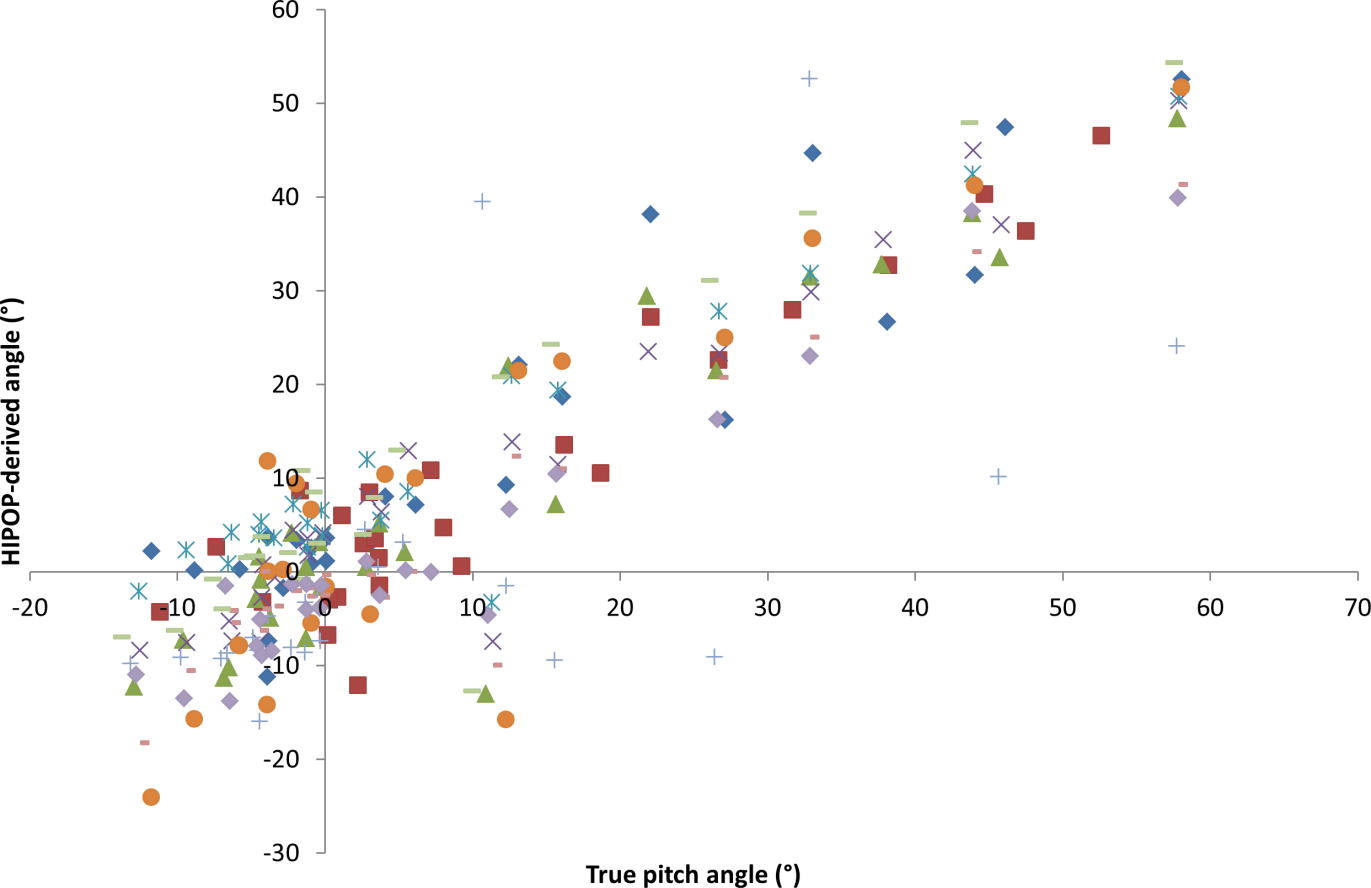
Head angle (A—pitch and B—heading) derived from the head mounted HIPOP in relation to the respective direct line of sight angles using data from participants (individuals shown by different symbols) asked to look at defined targets within a university quadrangle (see text for details).

Tests within the university quad showed that true pitch angle closely matched the HIPOP-derived angle with best fits being a linear regression (Fig. 3A—mean *r*^2^ across participants = 0.88, range 0.78–0.93, SD 0.045), with similar variance to the true heading angle and the HIPOP-derived heading angle (Fig. 3B—mean *r*^2^ = 0.98, range 0.95–0.99, SD 0.012).

#### Preliminary observations on patterns of head movement

**Figure 4:**
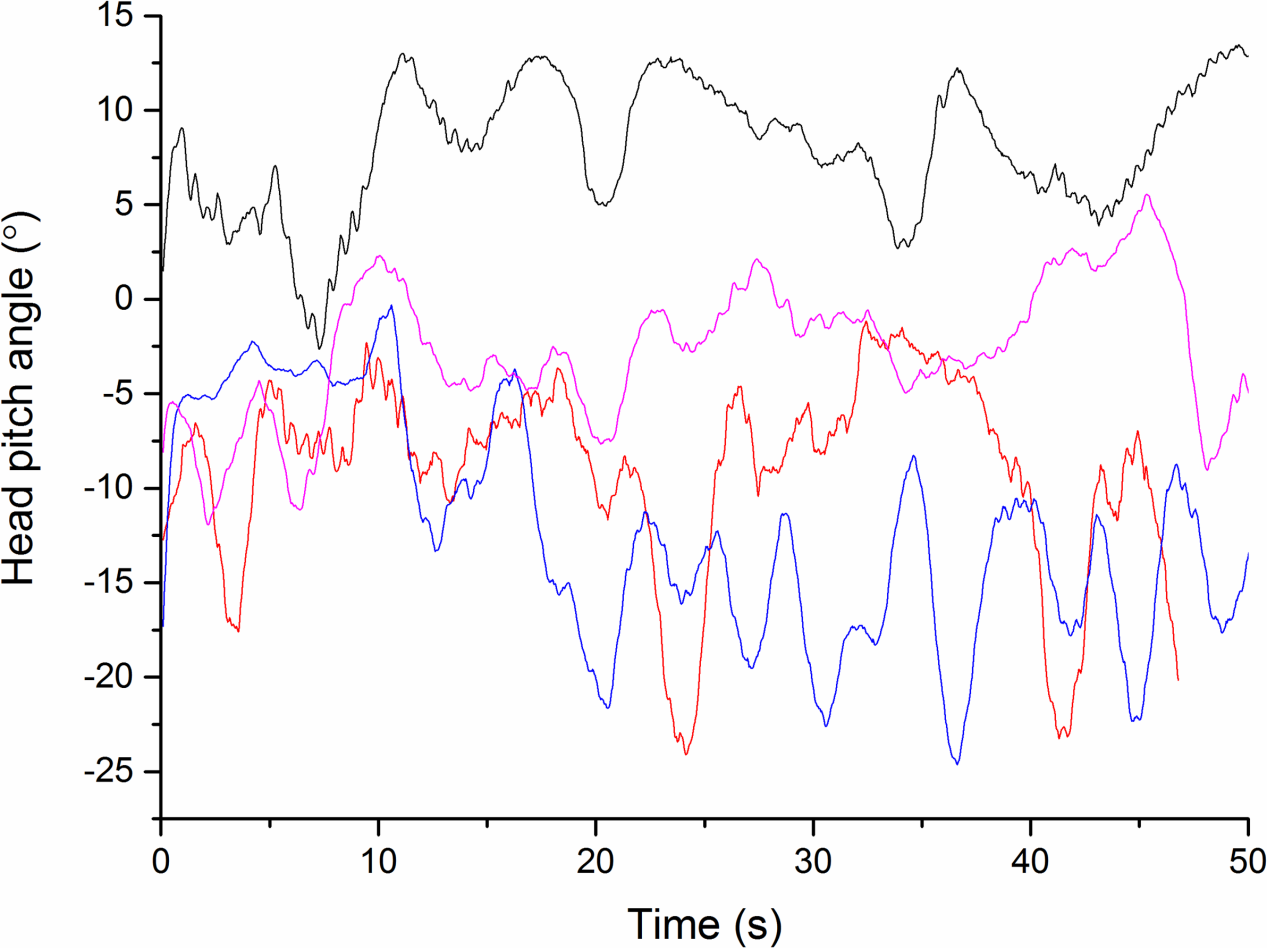
Head pitch angle from four different subjects (differently coloured lines) walking down an unmarked corridor. Note the small oscillations in the top trace due to individual strides.

**Figure 5:**
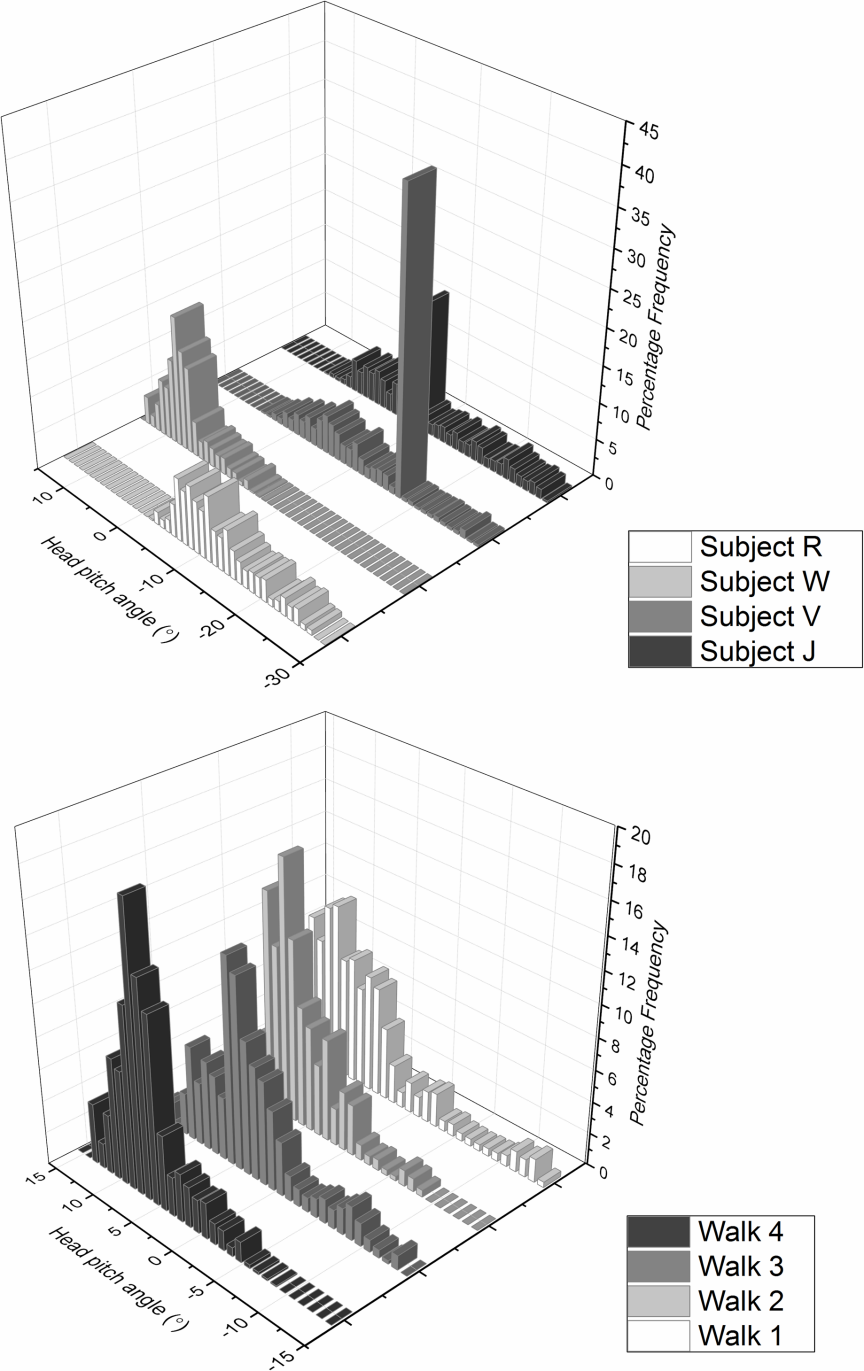
Frequency distribution of head pitch angle from (A) four differenr subjects and (B) the same subject (subject W) performing the task four times, walking down an unmarked corridor. The plots show marked inter-subject variation and minimal intra-subject variation.

**Figure 6:**
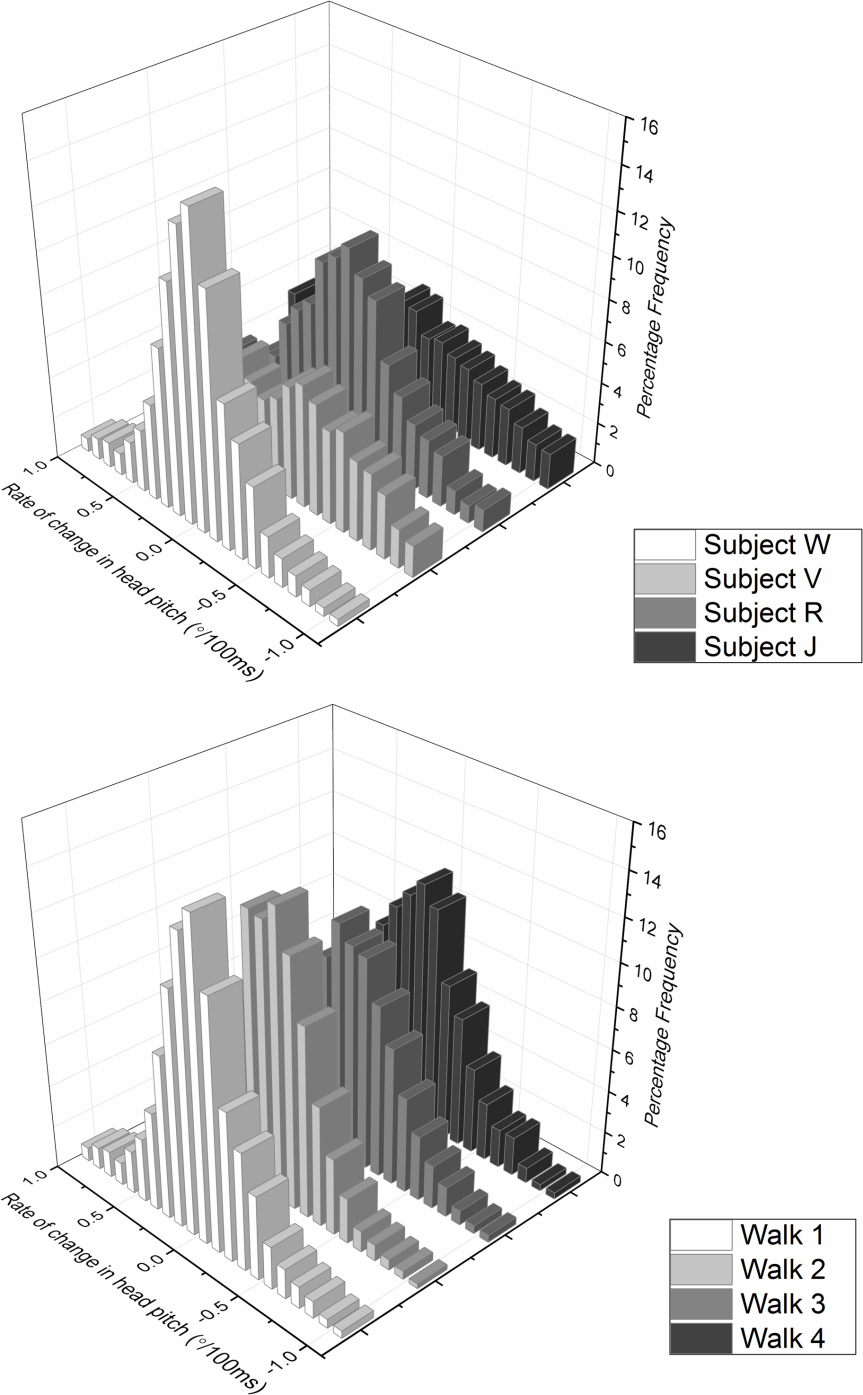
Frequency distribution of rate of change of head pitch angle from (A) four different subjects (same as Fig. 5—denoted by letters) and (B) the same subject (subject W) performing the task four times, walking down an unmarked corridor. As in Fig. 5, the plots show marked inter-subject variation and minimal intra-subject variation.

Data obtained from participants walking down a featureless corridor demonstrated stylised movement in subject head pitch even when environmental data were minimal. Typically the head pitch formed a wave motion with a wavelength of about 0.3 Hz, although there were marked differences between individuals (Fig. 4). In this task, it was notable that participants operated within individually specific ranges (Fig. 5A), with much greater between- than within-individual variation (Fig. 5B) and that this premise also held for the rate of change of head pitch angle (Fig. 6).

### Tests in outdoor setting

Data collected from people moving freely outside showed that participants spent a mean of nearly three hours (*x* = 2.95 h, SD = 0.83, *n* = 27) in the Garden, during which time they visited many of the major exhibits. However, a number of participants actually removed their hats (which was easily distinguishable by the head pitch and direction signals), rendering subsequent head pitch and heading values after this uncalibrated. For this reason, the following data refer only to the first 30 min after participants entered the garden and for a period when no hat adjustments were apparent. The information presented is intended to exemplify the sort of data that might be expected using the system in an outdoor setting rather than being a comprehensive treatise.

#### Head pitch and heading

**Figure 7:**
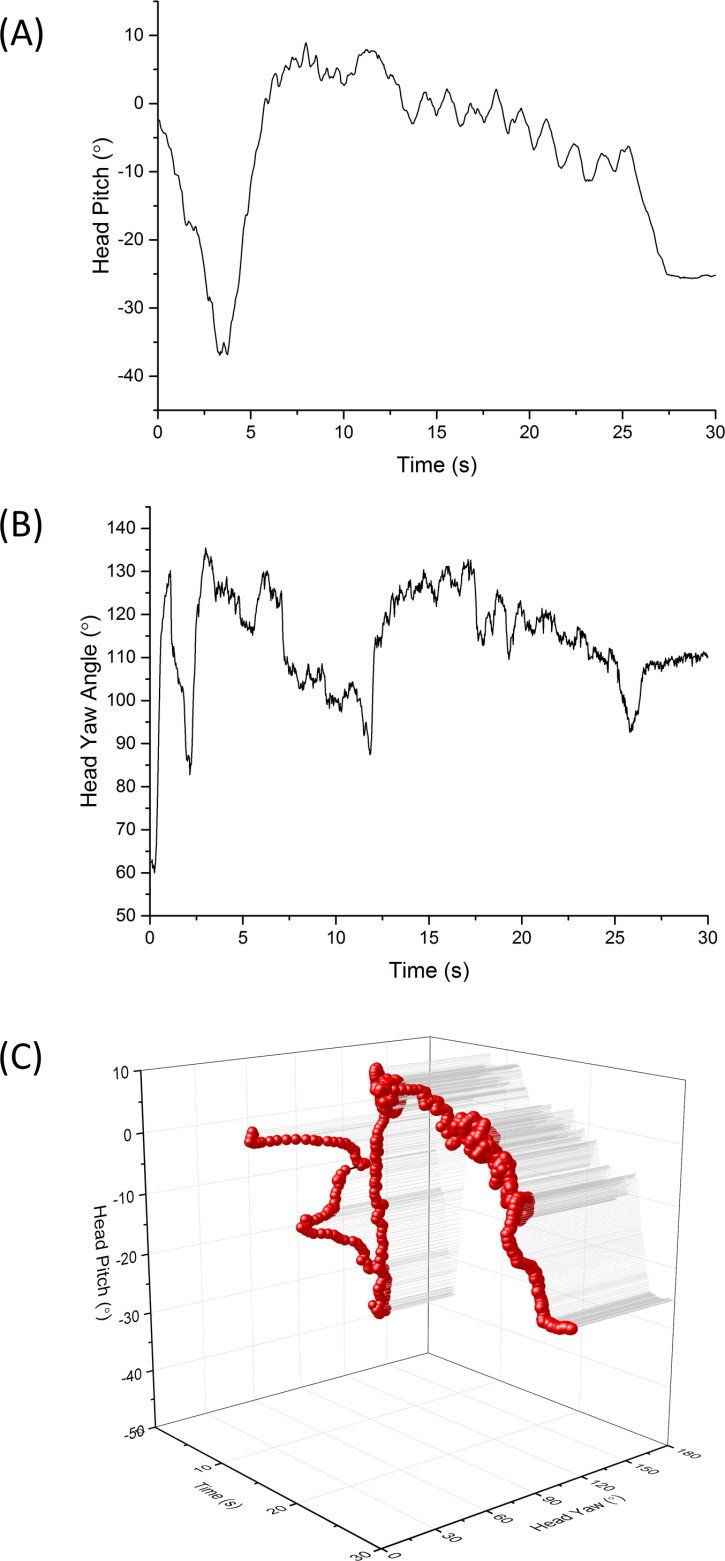
Example period of 30 s showing head directionality (A) pitch (B) heading and (C) both pitch and heading combined in a 3-dimensional plot, for a visitor walking through green space (the National Botanic Garden of Wales). Note the characteristic oscillations in pitch between about 12 and 25 s (cf. Fig. 4).

As with the participants indoors, those outside in the botanical garden exhibited a head movement behaviour that was characterised by constant movement in the pitch plane. When represented by a pitch *versus* time plot, head pitch typically showed waves of varying amplitude and wavelength (Fig. 7A). Head heading was similarly characterised by constant oscillations, also showing varying amplitude and wavelength (Fig. 7B) although waves were notably less symmetrical and less smooth than those in the pitch dimension (cf. Fig. 7A). The interaction of head pitch and head heading over time is shown in Fig. 7C.

**Figure 8:**
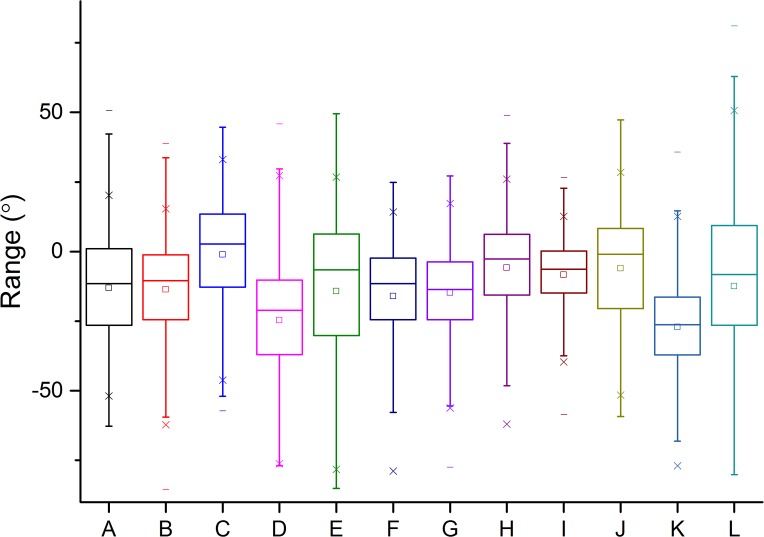
Box whisker plot of the head pitch angles of the different visitors (denoted by letters of the alphabet) to the National Botanic Garden of Wales during the first 30 min after entering the exhibit. Central points show means, boxes 25% confidence limits and vertical bars 95% confidence limits.

**Figure 9:**
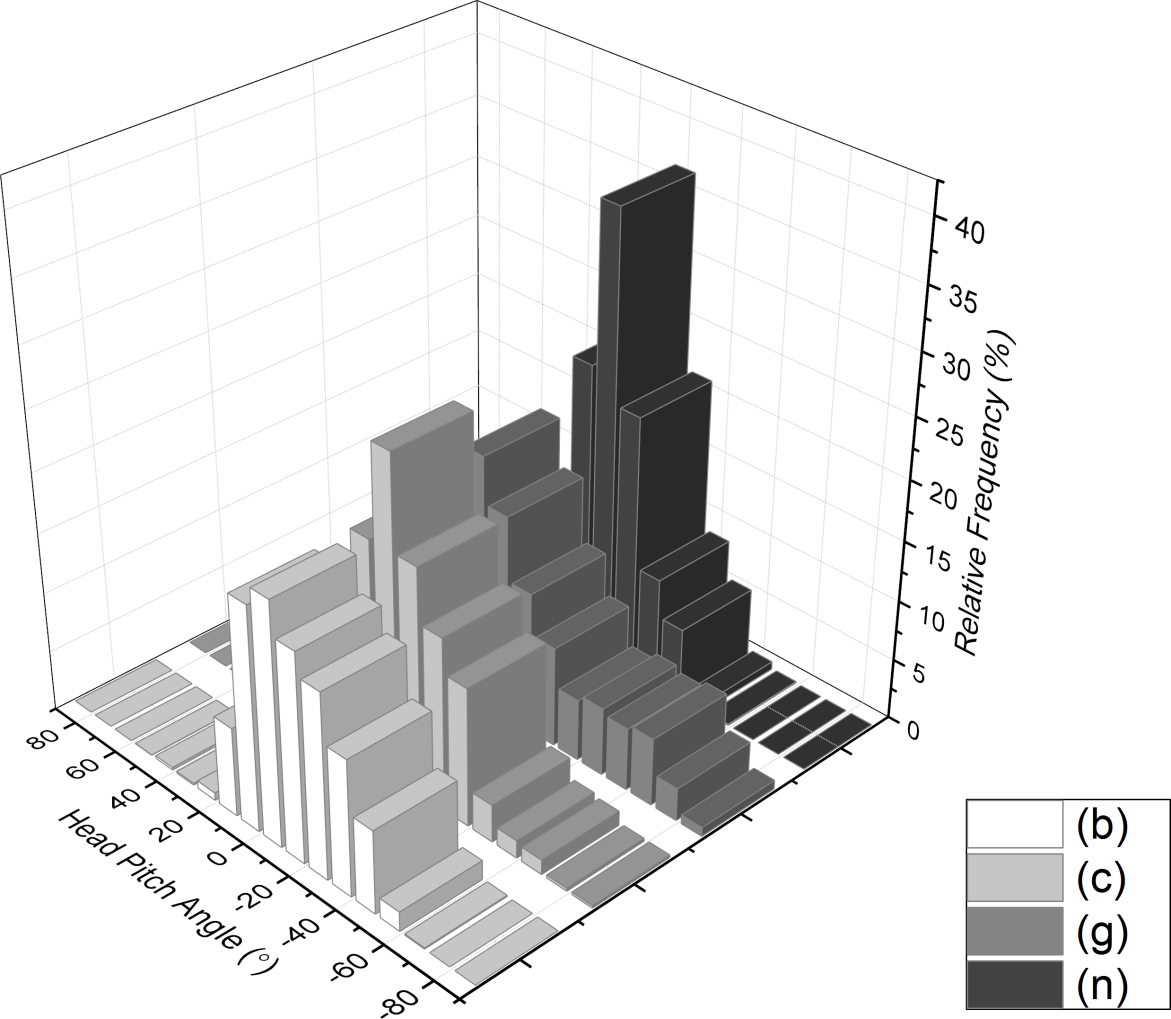
Frequency histogram of head pitch from example participants walking through the National Botanic Garden of Wales during the first 30 minutes after entry.

Overall, head pitch range was extensive with values between −80 and 60° for all participants, with most people exceeding 100° of arc (Fig. 8) although 25% confidence limits were restricted to about 25% (Table 1). The general picture was that people tended to have their head pitched down, with mean head pitch for participants ranging between −43° and 0° (equivalent modes were between −63° and 14° and medians between −39° and 4°). The source of this appreciable variation is apparent in frequency plots of head pitch, which generally showed a monomodal distribution, with an appreciable skew towards the head-down pitch attitudes (skewness values ranging between −1.3 and −0.1) (Fig. 9).

**Table 1:** Examples of metrics that can be derived from the HIPOP.

Metric	Unit
Instantaneous head pitch angle	°
Rate of change of head pitch angle	°/s
Head pitch angle distribution	°
Head pitch angle amplitude	°
Head pitch angle cycle duration	s
Head pitch fixation angle	°
Head pitch fixation duration	s
Instantaneous head heading	°
Rate of change of head heading angle	°/s
Head heading angle cycle amplitude	°
Head heading angle cycle duration	s
Head heading fixation angle	°

**Figure 10:**
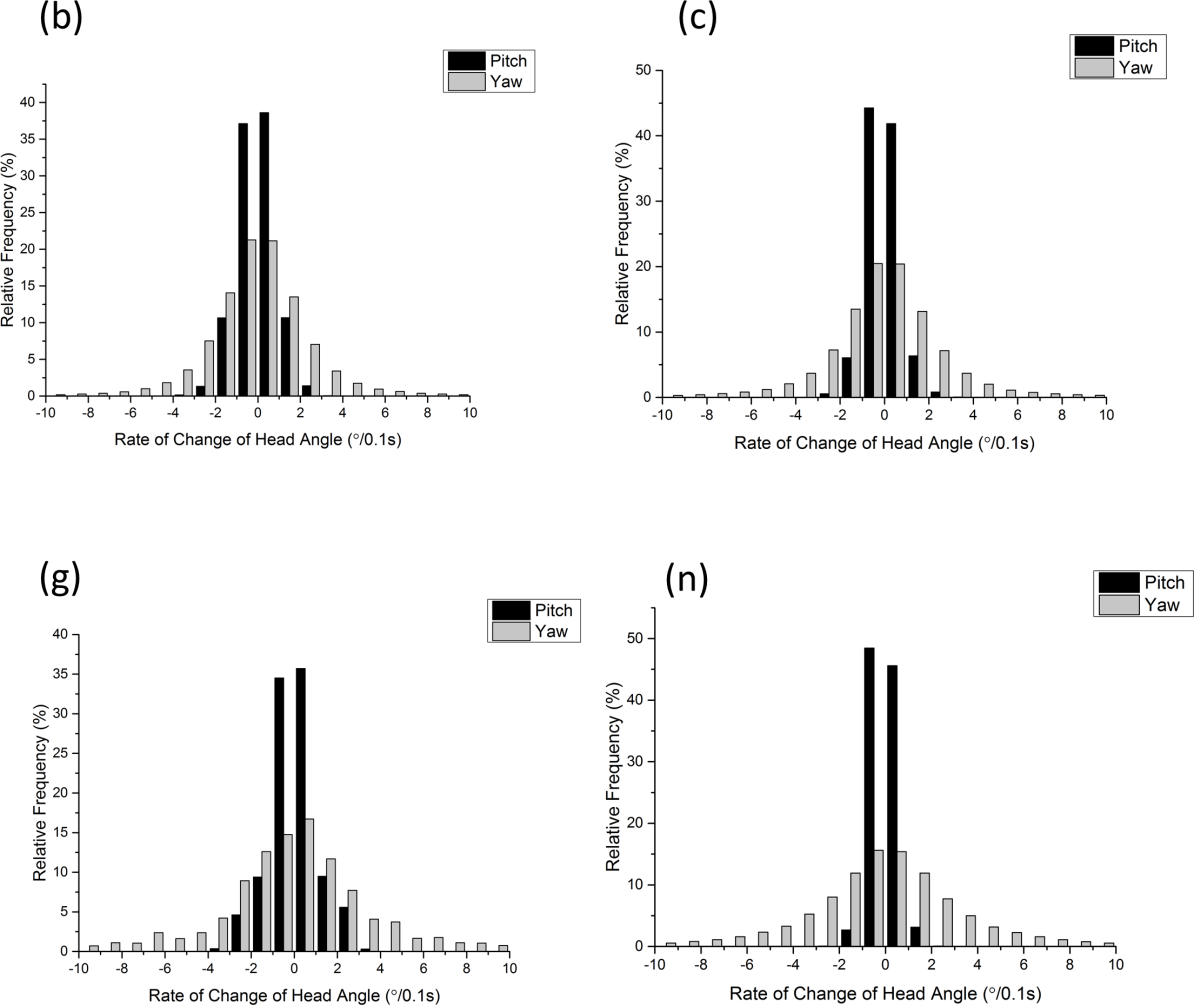
Frequency histograms showing rate of change of participants’ head pitch (black) and head yaw (grey) within the first 30 min enterering the National Botanic Garden of Wales (participants denoted by letters of the alphabet).

Mean rate of change of head pitch ranged from −0.00187°/0.1 s to 0.00187°/0.1 s (this short time interval was chosen for actual values to minimize the chances of head directionality changing during caculation), although this was more of a reflection of the stability of the head pitch than an expression of maximum rates of change of head pitch. Rates of change of head pitch are exemplified in the frequency distributions, which were symmetrical and monomodally distributed (Fig. 10).

**Figure 11:**
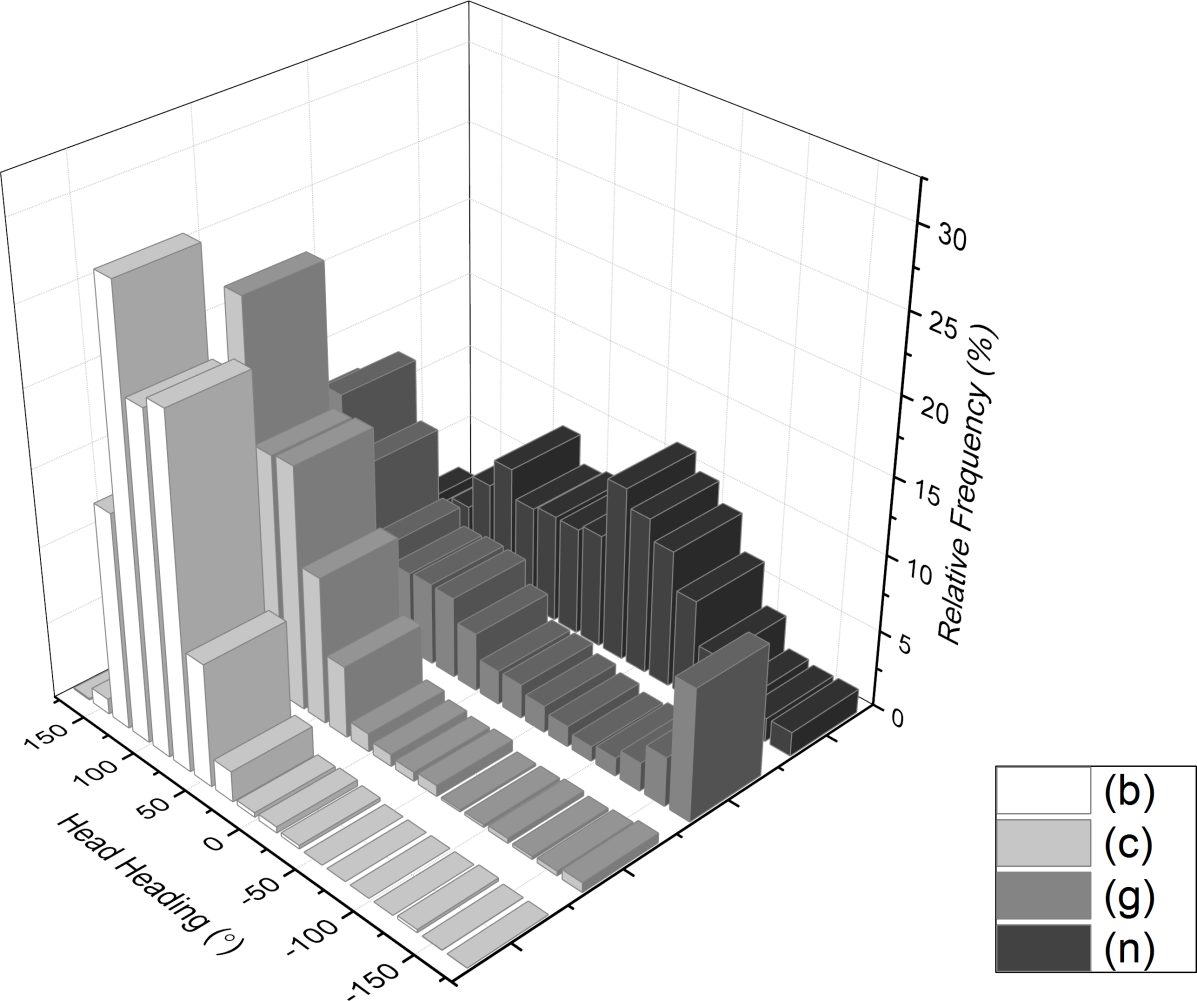
Frequency histogram of head heading (direction with respect to North = 0°) from example participants walking through the National Botanic Garden of Wales during the first 30 min after entry while they were walking in an approximately north-easterly direction (ca. 45°).

Unsurprisingly, head heading values varied more widely than head pitch since they covered the full 360°. For the period considered, participants showed appreciable variability in head heading (Fig. 11), with most participants having distributions that were distinctly non-normal.

Mean rate of change of head heading ranged from −0.3141 to 0.01263°/0.1 s. As with the head pitch, frequency distributions were symmetrical and monomodally distributed (Fig. 9).

**Figure 12:**
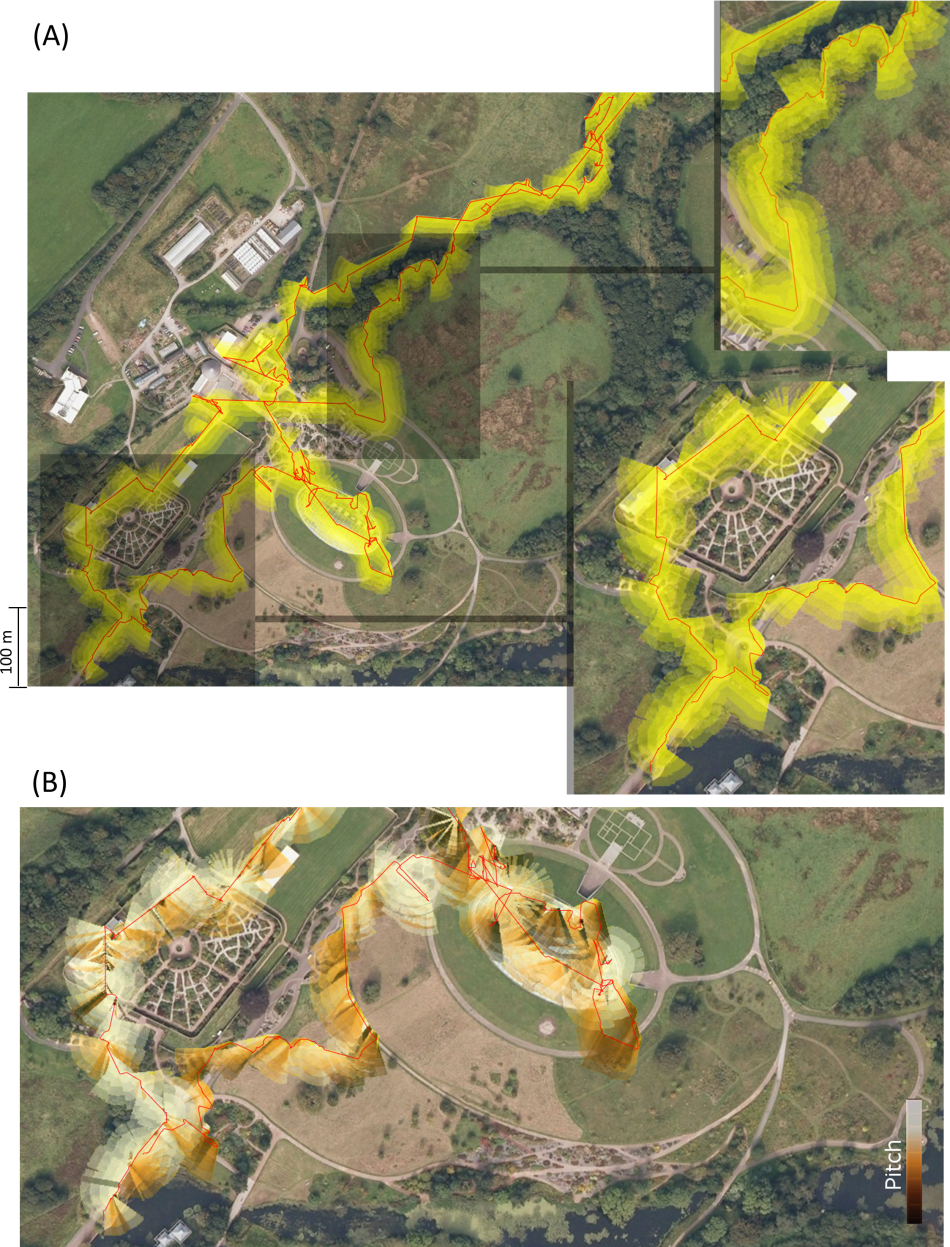
Visualisations of the trajectory of a visitor to the National Botanic Garden of Wales (red lines—determined using GPS) with head heading represented as lines extending from the trajectory. (A) shows variation according to locality and (B) emphasizes the changes in head pitch that occur over the course of the walk (pitch-down is darker colours, pitch up, the lighter ones).

The interplay of head pitch and head heading showed considerable variance along the paths taken by the participants in the green space of the botanical gardens, and highlighted role that head orientation might be expected to play in exposing people to certain features of the environment (Fig. 12).

## Discussion

### Head attitude and its role in perception of the environment

**Figure 13:**
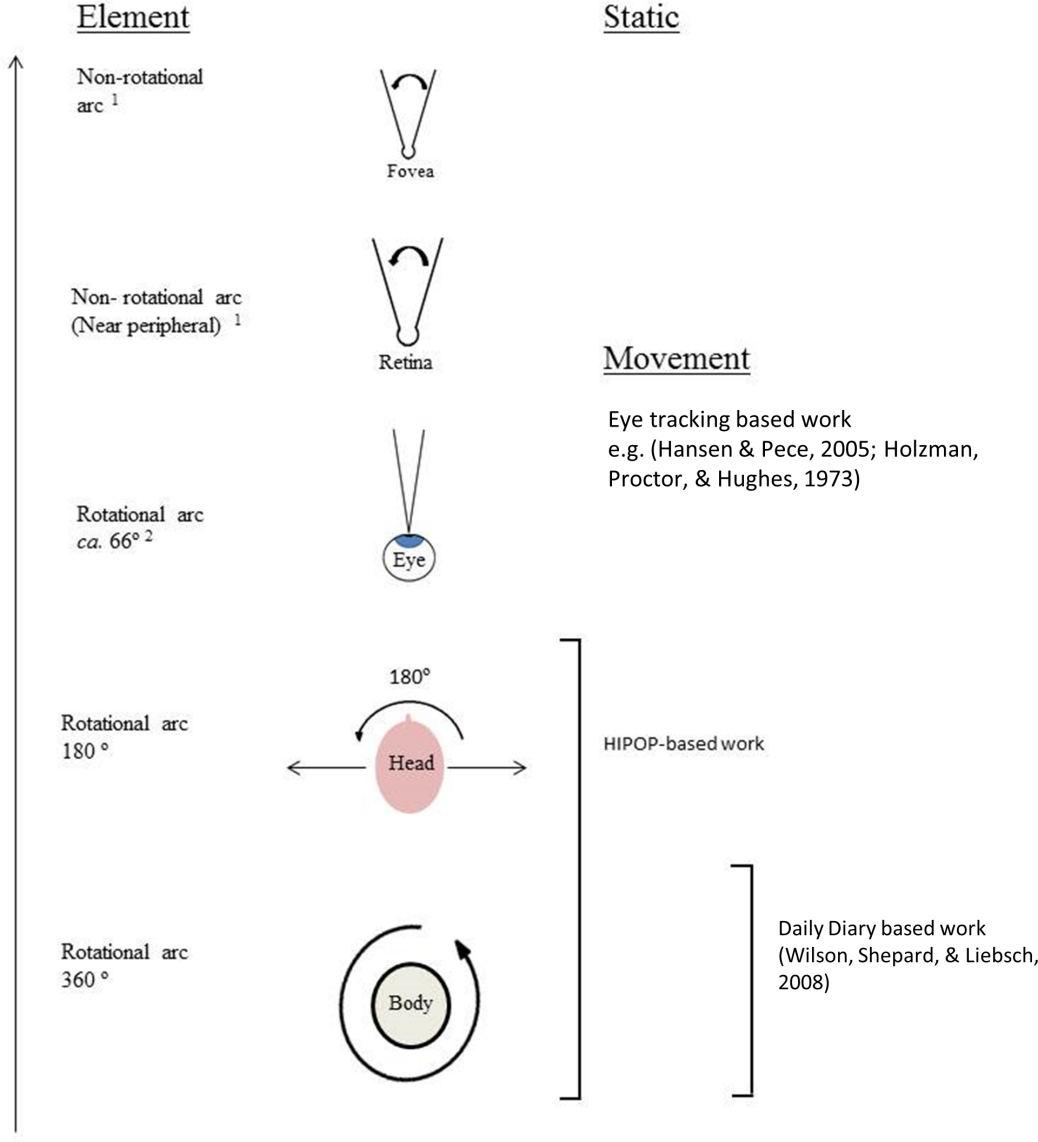
Hierarchy of rotating and non-rotating elements that determine the absolute head orientation and, beyond this, the elements that determine whether an object within the field of view afforded by the head orientation falls onto the fovea.

The attitude of the head is one of three movable elements that determine which part of the environment may fall as an image on the retina. After body orientation, which may cover up to the full 360° of both pitch and heading, the head attitude has a movement arc of almost 180° with respect to the body attitude (Fig. 13). Importantly, though, measurement of the head attitude, as done using the HIPOP here, gives information of the potential field of view for the eye retinas, which we term the ‘environmental frame.’ With any particular environmental frame, given by the head attitude, the six muscles controlling the movement of human eyes (Kolb, Fernandez & Nelson, 2007) allow eyes to scan this frame both horizontally and vertically. Thus, following definition of the environmental frame by the head, the movement limits of the eyes within their sockets give the range of possible gaze attitudes (c.f. Stahl, 1999). Although head and eye movement are closely coordinated (Morasso, Bizzi & Dichgans, 1973), with the implication that head attitude may give some indication of gaze attitude (cf. Fig. 3), the potential errors make the use of head attitude a much weaker indicator of gaze direction that eye trackers, for example (Spooner, Sakala & Baloh, 1980). Nonetheless, since the head attitude frames the environment for the eyes to within a defined cone, its quantification suggests that it may prove revealing with respect to the way we scan the environment generally as well as the features that influence the way in which we scan the environment.

### Head attitude quantification and behaviours expected to define the environmental frame

This non-exhaustive study clearly indicates that human head movement, as recorded by the accelerometer-magnetometer-based HIPOP system presented here, can be effectively quantified (although only if subjects do not remove their apparatus). There are two levels for which such data can be used; (i) as indices of behaviour, using metrics such head pitch and head heading over time (Fig. 7) and (ii) as an indicator of the features to which people expose their visual system.

Indices of behaviour, as manifest by head movement, are expected to be dependent on a suite of participant-dependent variables such as gender (c.f. Farenc, Rougier & Berger, 2003), age (c.f. Kauffman, 1987), social status (c.f. Cashdan, 1998) , and even state (c.f. Riskind & Gotay, 1982) as well as on the environment around the participant.

Overall though, head direction is likely to provide useful information on how humans react to, and position, the landscape with respect to their visual field: In other words, the head has to be orientated in a particular way for objects within the environment to be perceivable given the limitations on eye movement within the sockets. For instance, in other animals, there has been a considerable amount of work concerned with vigilance (e.g., Childress & Lung, 2003; Dimond & Lazarus, 1974; Lima & Bednekoff, 1999), where animals inspect the landscape for signs of predators and this behaviour has been derived from head movement (Treves, 2000). Although not quantified in terms of specifics such as the metrics that the HIPOP can provide, such work shows the importance in inspection of the landscape in animals, and it is even likely to occur in a general sense in humans, although perhaps not related specifically to predators (although motorized vehicles may be regarded as dangerous enough to quality for this).

If the head orientation as derived from the HIPOP is considered within this context, we can examine the value of head movements generally, especially as an adjunct to eye-scanning technologies (c.f. Guitton & Volle, 1987).

### General environmental framing

The expectation is that general landscape scanning would take the form of a very broad arc, perhaps spanning the full 360° at slow rates of change of heading. Because people tend to scan more in unfamiliar environments (Mourant & Rockwell, 1970), the percentage contribution of such head scan times might be expected to vary accordingly but the modalities of this are also expected to vary with travel speed (Hirasaki et al., 1999). General environmental framing is, however, liable to have a specific context such as to serve in navigation, facilitating movement or attention to specific elements of the environment and the nature of the function will define expected patterns in head orientation.

### Environmental framing for navigation

A part of assessment of the environment by moving humans will be specifically concerned with navigation and, since navigation by humans stems from vision, and specifically relating the position of the observer to landmarks (cf. Colin de Verdière & Crowley, 2000), we might expect head movement to display particular patterns related to this. Certainly, for eye tracking systems (Robinson & Taboada, 1994), it is expected that landmarks would be held as points of interest for some period (c.f. Becker & Fuchs, 1969) , although whether this can be simply translating into corresponding head movement given eye movement within the sockets as a confounding factor will need to be examined explicitly.

Putative head movements in conventional navigation may be further modified by the use of mobile phones, tablets or maps, which are known to modify navigation behaviour markedly (Ishikawa et al., 2008). Here, we might expect head movement to show an increase in extreme head pitched-down attitude, as people consult their source of information.

### Environmental framing to inform movement

A likely important element in head movement will be inspection of the terrain for effective movement, with people looking down to ensure surefootedness. We expect, where this occurs, head pitched-down behaviour to correlate tightly with travel direction because people will be looking where they are going. Identification of this behaviour could not occur definitively within this study due to the precision of the positional data from the GPS. However, a carefully organised protocol that incorporated a GPS-enabled dead-reckoning system (Wilson, Shepard & Liebsch, 2008) with proper metrics for movement speed derived from accelerometry values (Bidder, Qasem & Wilson, 2012; Bidder et al., 2012), should show how people orientate their heads to pitch down as part of the normal process of safe locomotion through the environment. Specifically, it is to be expected that the head heading should correlate tightly to body travel direction, that this correlation would be tightest at greater speeds (Land, 2004) and that the head would pitch down more as the terrain became more difficult. In addition, this terrain inspection for effective movement behaviour is likely to vary with age, with older people perhaps having a greater negative head pitch for a given environment, something that is expected to be related to their generally more depreciated sight (cf. Bressler, 1989) and instability (Sadeghi et al., 2004). Finally, light intensity is likely to affect the inspection of the terrain for effective movement behaviour, with diminished light levels resulting in a greater negative pitch as people inspect the ground in front of them more carefully under sub-optimal lighting conditions.

Finally, in a visit to a botanical garden, and perhaps as a normal part of human behaviour, it is to be expected that head attitude values should reflect the interest of the participant in specific features such as signage, flowers, statues and water bodies. The varied nature of such points is expected to result in correspondingly varied head pitch and yaw values but there are some generalizations that can be made. Firstly, if such inspection is made while the observer is stationary, then the frequency distributions of the resultant pitch and yaw values are both expected to take the form of peaks, with the width of the distributions perhaps varying with the size and distance of the object inspected (tighter distributions are expected with farther and smaller objects). If, however, inspection is made while the observer is moving, then the distance between the observer and the object of interest will critically affect the frequency distribution of head yaw because the head must have a higher angular velocity in the yaw plane for closer objects.

### Head movement during interpersonal interactions

Head position and movement is also used in communication, for example in the formation of affect (Tom et al., 1991) and as an aid in speech perception (Munhall et al., 2004), including nodding and shaking of the head to show assent and dissent (cf. Briñol & Petty, 2003). In addition, extensive work on verbal and non-verbal communication shows that people communicating with each other generally face each other (Ekman & Rosenberg, 1997) and are likely to angle their heads to help maintain eye contact (cf. Kleinke, 1986). Thus, aside from communication being, at times, an appreciable component of head movement behaviour, it might be expected to result in tall people tending to have a pitch-down attitude while shorter people have a pitch-up attitude. Such patterns would be further complicated by the directionality of gaze according to the content of the conversation (Adams Jr & Kleck, 2005).

### Head attitude in a non-framing context

In addition, head directionality does not always have to relate directly to the environment, most obviously when people have their eyes closed, such as in sleep or during introspection or ‘daydreaming’ (cf. Antrobus, Antrobus & Singer, 1964).

Where the HIPOP is to be used as an indicator of what people are looking at is more challenging because the viability of the system depends on the accuracy of the determination of the wearer’s location and the distance between the wearer and objects of interest. The accuracy of (non-differentially corrected) GPS positioning is of the order of several metres (Dussault et al., 1999) so this resolution is clearly inadequate if objects of visual interest are to be determined over short distances. However, they might fall within the vision cone if this distance is large enough. A potential solution to this is to derive position using a combination of GPS positions and dead-reckoning (Wilson, Shepard & Liebsch, 2008), whereby errors in both systems can be used to correct each other although this requires that HIPOP wearers also carry appropriate devices. It is notable, however, that GPS-enabled dead-reckoning is unlikely to be viable for subjects within buildings and in any case, the use of steel in construction (as well as the preponderance of magnetic field-producing units within buildings, such as electrical wiring and computers) will cause errors in the calculation of head heading due to distortion of the earth’s magnetic field. Otherwise, the angular extent of the vision cone will be a primary determinant of the system error for determining objects of interest, with this error increasing with distance between subject and object according to simple trigonometric rules. Thus, for a vision cone of around 9°, the diameter of the vision circle will be 0.16 m at a subject-object distance of 1 m, 1.6 m at 10 m and 31.5 m at 200 m. This situation is likely to be further complicated where eye residence times are short, which may result in greater line of sight error cones.

The general flexibility of the HIPOP, which is based on a device used in wild animal tracking (Wilson, Shepard & Liebsch, 2008), also means that it could be valuable in studies of human movement, and the extent to which movement patterns are related to the way humans orientate their heads to perceive the environment (as manifest by the HIPOP head movement metrics). In short, the HIPOP, does seem to lend itself to studies seeking to quantify both how people inform themselves about their environment, and how they react to what they see.

### Derived metrics

The HIPOP has potential for producing a suite of interesting metrics that could prove useful in defining how people inform themselves about their environment, which we broadly classify as ‘informing behaviour.’ Metrics describing the way people perceive the environment have been proposed in eye tracking studies and include measures based on fixation-related, saccade-related, scan-path-related and gaze-related parameters (Ehmke & Wilson, 2007) and include ‘long fixations,’ ‘scan path’ and ‘back-track saccades’ (Ehmke & Wilson, 2007). A non-exhaustive list of metrics that can be calculated from HIPOP environmental framing data is presented in Table 1, which displays the relatively large number of options which show considerable diversity, and perhaps comparable to eye-tracking studies, even if the specifics of gaze direction are not given. Aside from the obvious head orientation metrics, comparable, though not equivalent, to those derived from eye-tracking studies such as eye residence time (Galesic et al., 2008), and eye fixation rate (Russo & Rosen, 1975), we suggest that HIPOP-derived data could be used to examine whether there are particular patterns in the way people frame the environment. An example of this is provided by the very clear wave forms apparent in head pitch in subjects walking along a virtually featureless corridor (Fig. 4). Although it is beyond the scope of this work to highlight differences in metrics according to subject, or environmental conditions, we did note substantive differences in behaviour between participants navigating in a featureless corridor and outside, which suggests that examination of ‘informing behaviour’ will be an exciting avenue for future research.

### Potential areas of application

The implication from this research is that the HIPOP has a number of societally useful applications as well as a place in informing blue skies science. For instance, there may be practical uses linked to the study of people with visual field deficits, examples of which are tunnel vision (Peli et al., 2007), glaucoma (Bramley et al., 2008) or scotomas (Schuchard, 2005). These conditions are expected to produce very different head behaviours (although work would have to ascertain this) so that, for example, the progression of rehabilitation protocols (e.g., Gieser et al., 2006) could be assessed using the HIPOP to quantify accompanying changes in the way the environment is framed.

In a non-pathological context, the HIPOP could inform us of how people frame their environment according to height, gender, ethnicity, or age, allowing marketing efforts to be directed at those sites. This would provide a valuable feedback mechanism for outdoor exhibits such as zoos or botanical gardens. It should also be possible to examine which sort of features in the environment (colour, size etc.) are most likely to attract attention, inciting people away from their normal scan directives (cf. Fig. 4A), which is also likely to be important from a marketing perspective.

More fundamental, however, the manner in which people frame the environment is critically important for initiatives seeking to inform members of the public generally, such as signage in cities. It would be highly significant if we discovered, for example, that older people tend to look down (have a negative head pitch angle) and thus are poorly informed by city signage. We note that many investigations have suggested the impact of age on both head turning and gait (Isler, Parsonson & Hansson, 1997; Lockhart, Woldstad & Smith, 2003) as well as the relevance of reduced sight in elderly (Wormald et al., 1992). Facets such as this may help explain space use by sectors of the population. This is analogous to research on road user behaviour (drivers, cyclists and pedestrian behaviour), where studies able to identify where people look the least (and thus are most prone to accident) are indispensable, particularly now that technology is responsible for major distractions on the road (Nasar, Hecht & Wener, 2008).

With the human world changing faster now than it has done at any time in the past, the subject of how we inform ourselves is becoming ever more topical. Our very preliminary research indicates that head-mounted systems such as ours may provide us with a useful new tool with which to study the consequences of this and give us an idea as to how to mitigate those elements that are detrimental.

## Supplemental Information

10.7717/peerj.908/supp-1 Supplemental Information 1 Consent Letter for Figure 2

10.7717/peerj.908/supp-2 Supplemental Information 2 Consent Form

10.7717/peerj.908/supp-3 Supplemental Information 3 Raw data (a.)

10.7717/peerj.908/supp-4 Supplemental Information 4 Raw data (b.)

10.7717/peerj.908/supp-5 Supplemental Information 5 Raw data (c.)

10.7717/peerj.908/supp-6 Supplemental Information 6 Raw data (d.)

10.7717/peerj.908/supp-7 Supplemental Information 7 Raw data (e.)

10.7717/peerj.908/supp-8 Supplemental Information 8 Raw data (f.)

10.7717/peerj.908/supp-9 Supplemental Information 9 Raw data (g.)

10.7717/peerj.908/supp-10 Supplemental Information 10 Raw data (h.)

10.7717/peerj.908/supp-11 Supplemental Information 11 Raw data (i.)

10.7717/peerj.908/supp-12 Supplemental Information 12 Raw data (j.)

10.7717/peerj.908/supp-13 Supplemental Information 13 Raw data (k.)

10.7717/peerj.908/supp-14 Supplemental Information 14 Raw data (l.)

10.7717/peerj.908/supp-15 Supplemental Information 15 Corridor walk

## References

[ref-1] Adams RB, Kleck RE (2005). Effects of direct and averted gaze on the perception of facially communicated emotion. Emotion.

[ref-2] Antrobus JS, Antrobus JS, Singer JL (1964). Eye movements accompanying daydreaming, visual imagery, and thought suppression. The Journal of Abnormal and Social Psychology.

[ref-3] Arrington C, Carr T, Mayer A, Rao S (2000). Neural mechanisms of visual attention: object-based selection of a region in space. Journal of Cognitive Neuroscience.

[ref-4] Becker W, Fuchs A (1969). Further properties of the human saccadic system: eye movements and correction saccades with and without visual fixation points. Vision Research.

[ref-5] Bidder OR, Qasem LA, Wilson RP (2012). On higher ground: how well can dynamic body acceleration determine speed in variable terrain?. PLoS ONE.

[ref-6] Bidder OR, Soresina M, Shepard EL, Halsey LG, Quintana F, Gómez-Laich A, Wilson RP (2012). The need for speed: testing acceleration for estimating animal travel rates in terrestrial dead-reckoning systems. Zoology.

[ref-7] Bramley T, Peeples P, Walt JG, Juhasz M, Hansen JE (2008). Impact of vision loss on costs and outcomes in medicare beneficiaries with glaucoma. Archives of Ophthalmology.

[ref-8] Bressler S (1989). Health maintenance issues of the elderly. Vision: age-related macular degeneration. Maryland Medical Journal.

[ref-9] Briñol P, Petty RE (2003). Overt head movements and persuasion: a self-validation analysis. Journal of Personality and Social Psychology.

[ref-10] Cai J, Andersen NL, Malureanu C (2001). In-field practical calibration of three-axis magnetometers.

[ref-11] Cashdan E (1998). Smiles, speech, and body posture: how women and men display sociometric status and power. Journal of Nonverbal Behavior.

[ref-12] Cerrolaza JJ, Villanueva A, Cabeza R (2012). Study of polynomial mapping functions in video-oculography eye trackers. ACM Transactions on Computer-Human Interaction.

[ref-13] Childress MJ, Lung MA (2003). Predation risk, gender and the group size effect: does elk vigilance depend upon the behaviour of conspecifics?. Animal Behaviour.

[ref-14] Cleveland D, Cleveland JH, Norloff PL (1993). Eye tracking method and apparatus. http://www.google.com/patents/US5231674.

[ref-15] Colin de Verdière V, Crowley JL (2000). Local appearance space for recognition of navigation landmarks. Robotics and Autonomous Systems.

[ref-16] Cooke L (2005). Eye tracking: how it works and how it relates to usability. Technical Communication.

[ref-17] Dimond S, Lazarus J (1974). The problem of vigilance in animal life. Brain, Behavior and Evolution.

[ref-18] Duchowski AT (2002). A breadth-first survey of eye-tracking applications. Behavior Research Methods, Instruments, & Computers.

[ref-19] Dussault C, Courtois R, Ouellet J-P, Huot J (1999). Evaluation of GPS telemetry collar performance for habitat studies in the boreal forest. Wildlife Society Bulletin.

[ref-20] Ehmke C, Wilson S (2007). Identifying web usability problems from eye-tracking data.

[ref-21] Ekman P, Rosenberg EL (1997). What the face reveals: basic and applied studies of spontaneous expression using the Facial Action Coding System (FACS).

[ref-22] Fang L, Antsaklis PJ, Montestruque LA, McMickell MB, Lemmon M, Sun Y, Fang H, Koutroulis I, Haenggi M, Xie M (2005). Design of a wireless assisted pedestrian dead reckoning system-the NavMote experience. IEEE Transactions on Instrumentation and Measurement.

[ref-23] Farenc I, Rougier P, Berger L (2003). The influence of gender and body characteristics on upright stance. Annals of Human Biology.

[ref-24] Freescale Semiconductor (2012). Implementing a tilt-compensated eCompass using accelerometer and magnetometer sensors. http://cache.freescale.com/files/sensors/doc/app_note/AN4248.pdf.

[ref-25] Galesic M, Tourangeau R, Couper MP, Conrad FG (2008). Eye-Tracking data new insights on response order effects and other cognitive shortcuts in survey responding. Public Opinion Quarterly.

[ref-26] Gieser DK, Williams RT, O’Connell W, Pasquale LR, Rosenthal BP, Walt JG, Katz LM, Siegartel LR, Wang L, Rosenblatt LC (2006). Costs and utilization of end-stage glaucoma patients receiving visual rehabilitation care: a US multisite retrospective study. Journal of Glaucoma.

[ref-27] Guitton D, Volle M (1987). Gaze control in humans: eye-head coordination during orienting movements to targets within and beyond the oculomotor range. Journal of Neurophysiology.

[ref-28] Hansen DW, Ji Q (2010). In the eye of the beholder: a survey of models for eyes and gaze. IEEE Transactions on Pattern Analysis and Machine Intelligence.

[ref-29] Hirasaki E, Moore ST, Raphan T, Cohen B (1999). Effects of walking velocity on vertical head and body movements during locomotion. Experimental Brain Research.

[ref-30] Ishikawa T, Fujiwara H, Imai O, Okabe A (2008). Wayfinding with a GPS-based mobile navigation system: a comparison with maps and direct experience. Journal of Environmental Psychology.

[ref-31] Isler RB, Parsonson BS, Hansson GJ (1997). Age related effects of restricted head movements on the useful field of view of drivers. Accident Analysis & Prevention.

[ref-32] Jonas H (1954). The nobility of sight. Philosophy and Phenomenological Research.

[ref-33] Kauffman T (1987). Posture and age. Topics in Geriatric Rehabilitation.

[ref-34] Kleinke CL (1986). Gaze and eye contact: a research review. Psychological Bulletin.

[ref-35] Kolb H, Fernandez E, Nelson R (2007). Gross anatomy of the eye.

[ref-36] Land MF (2004). The coordination of rotations of the eyes, head and trunk in saccadic turns produced in natural situations. Experimental Brain Research.

[ref-37] Lima SL, Bednekoff PA (1999). Back to the basics of antipredatory vigilance: can nonvigilant animals detect attack?. Animal Behaviour.

[ref-38] Lockhart TE, Woldstad JC, Smith JL (2003). Effects of age-related gait changes on the biomechanics of slips and falls. Ergonomics.

[ref-39] Mishkin M, Ungerleider LG, Macko KA (1983). Object vision and spatial vision: two cortical pathways. Trends in Neurosciences.

[ref-40] Morasso P, Bizzi E, Dichgans J (1973). Adjustment of saccade characteristics during head movements. Experimental Brain Research.

[ref-41] Mourant RR, Rockwell TH (1970). Mapping eye-movement patterns to the visual scene in driving: an exploratory study. Human Factors: The Journal of the Human Factors and Ergonomics Society.

[ref-42] Munhall KG, Jones JA, Callan DE, Kuratate T, Vatikiotis-Bateson E (2004). Visual prosody and speech intelligibility: head movement improves auditory speech perception. Psychological Science.

[ref-43] Nasar J, Hecht P, Wener R (2008). Mobile telephones, distracted attention, and pedestrian safety. Accident Analysis & Prevention.

[ref-44] Peli E, Luo G, Bowers A, Rensing N (2007). Applications of augmented-vision head-mounted systems in vision rehabilitation. Journal of the Society for Information Display.

[ref-45] Rayner K (1998). Eye movements in reading and information processing: 20 years of research. Psychological Bulletin.

[ref-46] Riskind JH, Gotay CC (1982). Physical posture: could it have regulatory or feedback effects on motivation and emotion?. Motivation and Emotion.

[ref-47] Robinson W, Taboada J (1994). Eye tracking system and method. http://www.google.com/patents/US5345281.

[ref-48] Russo JE, Rosen LD (1975). An eye fixation analysis of multialternative choice. Memory & Cognition.

[ref-49] Sadeghi H, Prince F, Zabjek KF, Labelle H (2004). Simultaneous, bilateral, and three-dimensional gait analysis of elderly people without impairments. American Journal of Physical Medicine & Rehabilitation.

[ref-50] Schuchard RA (2005). Preferred retinal loci and macular scotoma characteristics in patients with age-related macular degeneration. Canadian Journal of Ophthalmology/Journal Canadien d’Ophtalmologie.

[ref-51] Shepard EL, Wilson RP, Halsey LG, Quintana F, Laich AG, Gleiss AC, Liebsch N, Myers AE, Norman B (2008a). Derivation of body motion via appropriate smoothing of acceleration data. Aquatic Biology.

[ref-52] Shepard EL, Wilson RP, Quintana F, Laich AG, Liebsch N, Albareda DA, Halsey LG, Gleiss A, Morgan DT, Myers AE (2008b). Identification of animal movement patterns using tri-axial accelerometry. Endangered Species Research.

[ref-53] Spooner JW, Sakala SM, Baloh RW (1980). Effect of aging on eye tracking. Archives of Neurology.

[ref-54] Stahl JS (1999). Amplitude of human head movements associated with horizontal saccades. Experimental Brain Research.

[ref-55] Tom G, Pettersen P, Lau T, Burton T, Cook J (1991). The role of overt head movement in the formation of affect. Basic and Applied Social Psychology.

[ref-56] Treves A (2000). Theory and method in studies of vigilance and aggregation. Animal Behaviour.

[ref-57] Walls GL (1942). The vertebrate eye and its adaptive radiation.

[ref-58] Wilson RP, Shepard E, Liebsch N (2008). Prying into the intimate details of animal lives: use of a daily diary on animals. Endangered Species Research.

[ref-59] Wormald R, Wright L, Courtney P, Beaumont B, Haines A (1992). Visual problems in the elderly population and implications for services. BMJ.

