# Supplementary material for: A new perspective on how humans assess their surroundings; derivation of head orientation and its role in ‘framing’ the environment
Source: PeerJ. 2015 Jun 18;3:e908. doi: 10.7717/peerj.908 (PMC4476166; doi:10.7717/peerj.908)
Supplement: Supplemental Information 1 [file peerj-03-908-s001.pdf]

Stet Oakley 6,
University of Reading,
Reading,
Berkshire,
RG6 6JE
18/03/2015

To whom it may concern,

I, Katherine Hinds, hereby permit the use of my photo depicting my face in the manuscript “A new perspective on how humans assess their surroundings; derivation of head orientation and its role in ‘framing’ the environment”.

Faithfully,

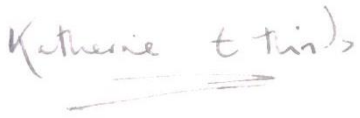

Katherine Hinds
