# Supplementary material for: A new perspective on how humans assess their surroundings; derivation of head orientation and its role in ‘framing’ the environment
Source: PeerJ. 2015 Jun 18;3:e908. doi: 10.7717/peerj.908 (PMC4476166; doi:10.7717/peerj.908)
Supplement: Supplemental Information 2 [file peerj-03-908-s002.docx]

**PARTICIPANT CONSENT FORM**

**Characterization of natural movement**

**Investigators:**  Gwendoline Wilson

**Biosciences, Swansea University**

This research will look at the behaviour of people visiting an outdoor exhibit with a view to defining the areas frequented by visitors and the specific points that attract most interest. This will be defined by tags fitted to hats worn by participants help determine how visitors move through the Garden.
The purpose of this study is to help the managers of the exhibit identify which features of the Garden are most valuable and appreciated by visitors.

“This study has been explained to me to my satisfaction, and I agree to take part. I understand that I am free to withdraw at any time without giving reason.”

Signature of the Participant: Date:

Name (in block capitals)

I have explained the study to the above participant and he/she has agreed to take part.

Signature of researcher: Date
